# Bioprospecting endophytic fungi for bioactive metabolites and use of irradiation to improve their bioactivities

**DOI:** 10.1186/s13568-022-01386-x

**Published:** 2022-04-19

**Authors:** El-Sayed R. El-Sayed, Magdia A. Hazaa, Magdy M. Shebl, Mahmoud M. Amer, Samar R. Mahmoud, Abeer A. Khattab

**Affiliations:** 1 Plant Research Department, Nuclear Research Center, Egyptian Atomic Energy Authority, Cairo, Egypt; 2 Biological Applications Department, Nuclear Research Center, Egyptian Atomic Energy Authority, Cairo, Egypt; 3 Department of Botany, and Microbiology Faculty of Science, Benha University, Benha, Qalubiya Governorate, Egypt

**Keywords:** Bioactives, Antioxidant, Antifungal, Antibacterial, Endophytes, Anticancer

## Abstract

The search for new bioactive compounds with innovative modes of action and chemistry are desperately needed to tackle the increased emergence of drug-resistant microbes. With this view, this paper was conducted for the isolation, identification, and biological evaluation of fungal endophytes of eleven different plant species. A total of 69 endophytic strains were isolated and tested for the presence of bioactive metabolites with antifungal, antibacterial, anticancer, and antioxidant properties in their extracts. Upon screening, two promising strains were found to have all the before-mentioned activities. These strains were *Aspergillus sydowii* isolated from the bark of *Ricinus communis* and *Aspergillus flavus* isolated from the twigs of *Psidium guajava*. Major compounds present in extracts of the two strains were identified by GC-Mass analyses. Several well-known bioactive compounds as well as unreported ones were identified in the fungal extracts of the two strains. Furthermore, gamma irradiation (at 1000 Gy) of the fungal cultures resulted in improved bioactivities of extracts from the two strains. These findings recommend the two fungal strains as sources of antimicrobial, anticancer, and antioxidant compounds which may aid in the development of novel drugs. The presented research also explains the high-value of fungal endophytes as untapped sources of bioactive metabolites.

## Keypoints

- Discovery of two promising fungal endophytes with divers’ range of bioactivities
- Extracts of the two strains showed antimicrobial, anticancer, and antioxidant activities
- Exposure to gamma rays at 1000 Gy significantly enhanced all the bioactivities.

## Introduction

Natural bioactive compounds are considered the cornerstone in the development of high-value products. Their bioactivity has supported their applications in medicine, agriculture, and the food industry (Atanasov et al. 2021). The search for new bioactive compounds and the study of their potential biological activities has emerged as one of the most promising and ambitious developments in science (Karthikeyan et al. 2022). For example, there is a continuous need for new antibiotics due to the emergence of resistant microbes, and a global need for other drugs to target unmet clinical needs for a range of diseases (Miethke et al. 2021). The resistant microbes are potential threats to human and animal health with serious consequences. Hence, new natural compounds must be identified and developed now, more than ever, to meet this urgent and growing demand for novel drugs.

In natural products discovery programs, bioprospecting plants for novel bioactive compounds is ongoing, and such sources of bio-based products, represented a market share of 47% (Newman and Cragg 2016). However, using plants as a source of complex therapeutics brings with its drawbacks including low yields and sustainable supply, frequently leading to over-harvesting. The use of microorganisms instead of the plants themselves has emerged as a promising strategy for offering compounds with high therapeutic potential (Alvin et al. 2009). The utilisation of microbial communities, especially fungi, has several advantages which rendered it more robust than other strategies. For instance, the culture medium for fungal growth and metabolism is relatively cheap and simple, so, cells have gained remarkable attention because of their promising biotechnological potential and diversity (Janso and Carter 2010). Recent scientific efforts have been aimed at the bioprospection of the endophytic fungi (fungal community inside plant tissues) for the isolation of new antimicrobials and anticancer drugs (Kumar and Mongolla 2018; Saha et al. 2019). Endophytic fungi have been studied mainly as sources of new bioactive compounds and secondary metabolites of their host plants with several applications in medicine, agriculture, and food industry (dos Santos et al. 2021; Mousa et al. 2021), as they proved to be untapped pools of novel bioactive molecules (Venugopalan and Srivastava 2015).

It is now known that most plants contain endophytic fungi that grow in plant tissues and aid nutrient assimilation, produce growth promoting metabolites, insect and pest repellents, but also antimicrobials which are indispensable for plant survival (Baron and Rigobelo 2022). As a consequence of co-evolutionary processes, these endophytes harbor an enormous metabolic potential to synthesize compounds with high bioactivity that can be exploited commercially (Nisa et al. 2015; and references therein). Moreover, they showed higher metabolic activity than their free counterparts (El-Sayed 2021). It is worthy here to mention that the possibility to find novel biologically active secondary metabolites from endophytic fungi is tremendous. For all of these reasons, we aim in this paper to unfold the untapped potential of endophytic fungi of some plant species as sources for bioactive compounds. Today, the bioprospecting research still needs much attention because few products from it are available in the pharmaceutical market (dos Santos et al. 2021). Moreover, several reports recommended the use of gamma rays as a physical mutagen to improve microbial cultures thereby developing hyper-producers of the bioactive metabolites of industrial significances (Parekh et al. 2000; El-Sayed 2021; Zaki and El-Sayed 2021). In this context, 69 fungal strains were isolated from different plant tissues and screened for the presence of bioactive metabolites with antifungal, antibacterial, anticancer, and antioxidant properties. GC–MS analysis of the fungal extracts was accomplished to identify the chemical constituents. Furthermore, the effect of gamma irradiation on bioactivities of extracts from the two strains was also adopted.

## Materials and methods

### Plant materials and isolation of fungal endophytes

Samples were removed by a sterile sharp blade from healthy plant parts. Table 1 presents the used plant species. Plant species were identified from different cultivated locations in Egypt. The collected plant samples were transported to the laboratory and used for isolation of fungal endophytes according to El-Sayed et al. (2020a). Samples were fragmented to small parts, successfully dipped in 70% ethanol (for 1 min) then 0.1% HgCl_2_ (for 1 min) to sterilize their surfaces, washed in sterile dist. water, and let to dry on a sterile filter paper. All parts were transferred aseptically to on the surface potato-dextrose (amended with streptomycin and tetracycline) agar plates and were incubated at 25 °C, then checked daily. The isolated cultures were checked for purity and stored in glycerol (15%) as a suspension of spores and mycelia at – 4 °C.

**Table 1:** Host plants, isolated endophytic fungal genera, and antifungal, antibacterial, anticancer, and antioxidant activities of their crude extract

Host plant		Endophytic fungal genera							
		*Aspergillus*	*Alternaria*	*Fusarium*	*Trichoderma*	*Lasiodiplodia*	*Acremonium*	*Cladosporium*	*Chaetomium*
*Azadirachta indica*	***L***	–	1	–	–	–	1^O^	–	–
	***T***	–	–	–	–	–	–	1	–
	***B***	–	–	1	1	–	–	–	–
*Ricinus communis*	***L***	–	–	–	–	–	–	–	–
	***T***	–	1	–	1	–	–	–	–
	***B***	1^F^^,B,C,O^	–	1	1	–	–	–	–
*Hibiscus rose-sinensis*	***L***	–	–	1	–	–	–	–	1
	***T***	1	1	–	1	–	–	–	–
*Psidium guajava*	***L***	–	1	–	–	–	–	–	–
	***T***	1^F^^,B,C,O^	–	1	–	–	1	1^C,O^	–
	***B***	1	1	–	1	–	–	1	–
*Malus domestica*	***L***	1	1	–	–	–	–	–	1
	***T***	–	–	–	1^O^	–	–	1	
	***B***	–	1	–	–	–	–	–	–
*Citrus medica*	***L***	1	1^B^	–	–	–	1	1	–
	***T***	–	–	–	–	–	–	–	–
	***B***	1	1	1	–	–	–	–	1^F^^,B^
*Olea europaea*	***L***	–	1	–	–	–	–	–	–
	***T***	–	1	1	–	–	1	–	–
	***B***	–	–	1	1	–	–	1^O^	–
*Cupressus sempervirens*	***L***	–	1	1	1	–	–	–	–
	***T***	–	1	–	–	–	1	–	1
	***B***	–	1	1	1	–	–	–	–
*Pinus sylvestris*	***L***	1^F^^,B^	1	–	1	–	–	–	–
	***T***	–	–	–	–	–	1	–	–
	***B***	–	1	–	1	–	1^B^		–
*Mangi feraindicia*	***L***	–	1^F^^,B^	–	–	–	–	1	–
	***T***	–	1	–	–	–	–	–	–
	***B***	–	–	–	1	–	–	–	–
*Terminalia arjuna*	***L***	–	1	–	1^F^^,B^	–	–	–	–
	***T***	–	1	–	–	–	–	–	–
	***B***	–	–	–	–	1	–	–	–
Total		8	20	9	13	1	7	7	4

PDA was used for endophytic fungi isolation

*T* Twig, *L* Leaf and *B* Bark

Fungal cultures were incubated for 7 days at 30 ℃

^F^Indicates antifungal activity, ^B^Indicates antibacterial activity, ^C^Indicates anticancer activity, and ^O^Indicates antioxidant activity. Nystatin and Amoxicillin/Clavulanic acid were used as standard antifungal and antibacterial at a concentration of 100 µg mL^−1^

### Cultivation conditions and preparation of fungal culture extracts

Spore suspension for each fungal isolate was harvested from 7-days old culture and the final spore concentration was counted and adjusted to 10^6^ mL^−1^ using a hemocytometer. 1 mL of this suspension was inoculated under aseptic conditions to 250 Erlenmeyer flasks containing 50 mL PD broth and incubated at 30 ℃ for 7 days.

After incubation, culture filtrate of each fungal isolate was extracted thrice with an equal amount of methylene chloride. The methylene chloride layers were separated by a separating funnel, pooled, passed over anhydrous sodium sulphate to remove any water. The methylene chloride layers were concentrated by evaporation using an IKA, RV10, Germany rotary evaporator under reduced pressure. The resultant dry film was carefully dissolved in HPLC-grade methanol and used for testing the antibacterial, antifungal, anticancer, and antioxidant activities.

### Screening bioactivities of the isolated endophytes

The prepared crude extracts of the isolated endophytic fungi were used to evaluate their antibacterial, antifungal, anticancer, and antioxidant activities, as follows:

**Antifungal and antibacterial sensitivity tests** Antifungal and antibacterial potentials of the extracts were investigated according to Pongtharangkul and Demirci (2004) using the agar-well diffusion assay. The antifungal assay was against two human pathogens *Aspergillus brasiliensis* ATCC16404 and *Candida albicans* ATCC10231. Under the same conditions, a positive control of Nystatin (standard antifungal) and methanol only were also applied. Meanwhile, the antibacterial was evaluated against different human pathogenic bacteria* Escherichia coli* ATCC11229 and *Staphylococcus aureus* ATCC6538. Under the same conditions a positive control of Amoxicillin/Clavulanic acid (standard antibiotic) was applied. After incubation, zones of inhibition were measured carefully.

**Antioxidant activity** Crude extracts of the all the fungal isolates were tested for their antioxidant activities by the free radical scavenging assay according to the method described by Thaipong et al. (2006) performed using 2,2^\^-diphenyl picrylhydrazyl (DPPH, Sigma-Aldrich, St. Louis, MO, USA). Simultaneously, a positive control of ascorbic acid (Sigma-Aldrich, St. Louis, MO, USA) was also tested. Scavenging activity (%) was calculated as the change in the absorbance of the mixture (DPPH + fungal crude extract) with respect to the DPPH solution only (control).

**Anticancer activity** Cytotoxicity was evaluated against human breast (MCF-7) adenocarcinoma by the sulforhodamine B (SRB) assay according to Skehan et al. (1990). 100 μL of the cell suspension (5 × 10^3^ cells) were transferred into 96-well plates then incubated for 24 h in complete media. These cells were treated with another 100 μL media containing the fungal extract. Cells were fixed after 72 h of exposure by replacing media with 10% Trichloroacetic acid (150 μL) and incubated for 1 h at 4 °C. The trichloroacetic acid solution was removed and the cells were washed 5 times with sterile deionized water. Then, 70 μL of 0.4%, w/v SRB solution was added and darkly-incubated for 10 min at room temperature. Plates were washed thrice with 1% acetic acid and let to air-dry overnight. Finally, 150 μL of TRIS (10 mM) solution was added to dissolve protein-bound SRB stain and the absorbance was measured at 540 nm (BMG LABTECH®-FLUOstar Omega microplate reader, Ortenberg, Germany). Cell viabilities was determined by the following equation:

% of Viability = [Absorbance of treated cells / Absorbance of control cells] × 100.

### Fungal strains

Sixty-nine endophytic fungal isolates were screened for their bioactivity (as described earlier). Among the isolated fungi, methylene chloride extracts of two different isolates were found to have antifungal, antibacterial, anticancer, antioxidant activities. These strains were *Aspergillus sydowii* isolated from the bark of *Ricinus communis* and *Aspergillus flavus* isolated from the twigs of *Psidium guajava*. The two respective strains were identified and deposited under numbers AUMC14506 and AUMC14507 in the Culture Collection (aun.edu.eg/aumc/aumc.htm) of Assiut University Mycological Center, Egypt.

### Identification of the selected endophytic fungi

Identification of the two strains was accomplished by colony morphology, growth characteristics, and molecular characterization. Morphological identification was performed by studying the colony on Czapek's-yeast autolysate agar according to Moubasher (1993). Czapek's-yeast autolysate agar composition (g L^−1^) as follows yeast extract 5, NaNO_3_ 3, sucrose 3, MgSO_4_.7H_2_O 0.5, KH_2_PO_4_ 0.5, KCl 0.5, and FeSO_4_.7H_2_O 0.01.

Molecular characterization was performed according to the method by White et al. (1990) using PCR-amplified ITS1-5.8S-ITS2 rRNA-gene. In brief, DNA of the fungal strains were extracted and sequenced by Solgent Company (Daejeon, South Korea). Sequences of the two strains were submitted to the GenBank and accession numbers were received. Finally, sequences were analyzed using the online tool (http://www.ncbi.nlm.nih.gov/) BLAST and the software BioEdit (version 7.0.1). A neighbor-joining tree with the maximum-likelihood for each fungal strain were constructed using MEGA software version 6.0.

### GC–MS analysis of the fungal extracts:

The methanolic extracts from the two fungal strains were analyzed using a Thermo Scientific, Trace GC Ultra/ISQ Single Quadrupole MS, TG-5MS fused silica capillary column (30 m, 0.251 mm, 0.1 mm film thickness). An electron ionization system (ionization energy of 70 eV) and Helium as the carrier gas (at a constant flow rate of 1 mL min^−1^) were used. The MS transfer line and the injector temperature were set at 280 ℃. Identification of all the compounds was performed by the comparison of their mass spectra and relative retention time with those of the NIST, WILLY library data of the GC–MS system.

### Influence of several ^60^Co gamma irradiation doses on the antifungal, antibacterial, anticancer, antioxidant activities

Spore suspensions of *Aspergillus sydowii* and *Aspergillus flavus* were prepared as described earlier. The two suspensions were separately irradiated by 250, 500, 1000, 2000 and 4000 Gy of gamma rays. A ^60^Co Gamma chamber, MC20, Russia (dose rate of 432.80 Gy h^−1^) was used. After irradiation, suspensions of each irradiation dose were kept in darkness overnight. An aliquot of 1 mL of each suspension was inoculated to 250-mL Erlenmeyer flasks (containing 50-mL potato-dextrose broth adjusted to pH 6.0) and incubated at 30 °C. Finally, fungal cultures corresponding to every irradiation dose were extracted concentrated according to the previous procedures. The antifungal, antibacterial, anticancer, antioxidant activities were estimated from each radiation dose, as described earlier.

### Statistics

The recorded results were expressed as the mean taken from triplicate measurements from three independent experiment ± the standard deviation. Statistical significance was studied by the ANOVA-test followed by Dunken’s test using as SPSS software (v. 22, IBM, NY) at 95% confidence intervals.

## Results

### Isolation and screening of potential fungal endophytes

Eleven different plant species were used to isolate endophytic fungi (Table 1) from different plant parts including twigs, leaves, and bark. A total of 69 fungal isolates were recovered from the plant samples on PD agar plates. The isolated endophytes were subjected to a preliminary morphological examination to identify their genera. Consequently, eight genera were recorded including *Aspergillus*, *Acremonium*, *Cladosporium*, *Trichoderma, Fusarium*, *Lasiodiplodia, and Alteranria*.

**Fig. 1:**
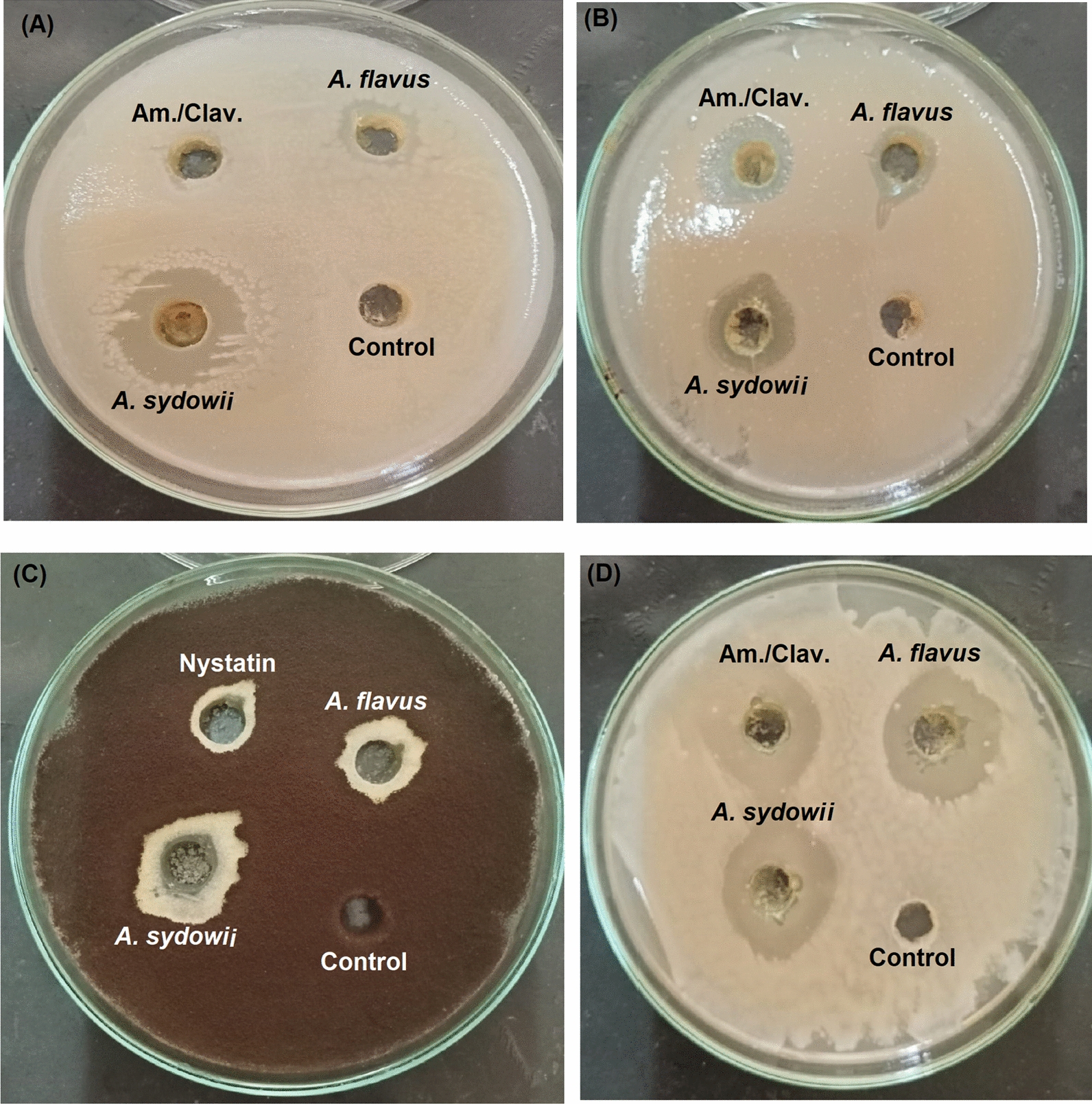
Photographs of the antimicrobial activity of *A. sydowii* AUMC14506 and *A. flavus* AUMC14507 crude extracts against *E. coli* (**A**), *S. aureus* (**B**), *A. brasiliensis* (**C**), and *C. albicans* (**D**)

**Fig. 2:**
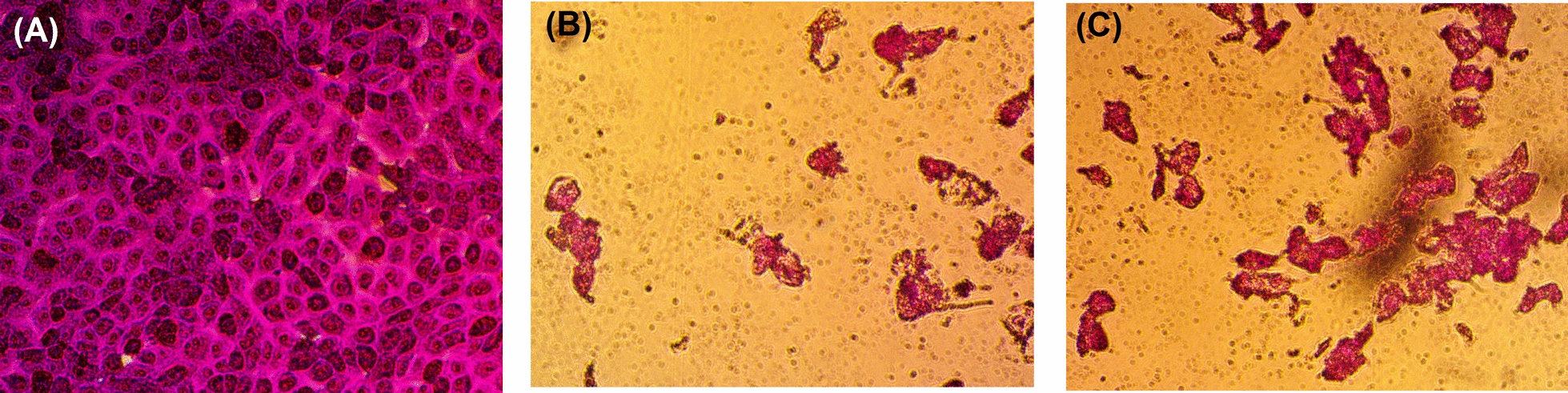
Photograph of the cytotoxic activity of *A. sydowii* AUMC14506 and *A. flavus* AUMC14507 crude extracts against human breast adenocarcinoma. **A** Control cancer cells, **B** Cells treated with extract of *A. sydowii*, and **C,** Cells treated with extract of *A. flavus*

The 69 isolates were separately cultured in potato-dextrose broth for 7 days at 30℃ then the culture broth was extracted. All the crude extracts from the isolated fungi were separately tested for their antifungal (against *A. brasiliensis* and *C. albicans*), antibacterial (against *S. aureus* and *E. coli*), anticancer (against MCF-7 human breast cancer), and antioxidant activities. Screening profile of the 69 isolates presented in Table 1 revealed that 12 strains showed varied activities. Among the 11 strains, only two strains were positive regarding all the tested activities. The two isolates showed all the tested bioactivates of antibacterial (Fig. 1a, b), antifungal (Fig. 1c, d), anticancer (Fig. 2), and antioxidant. Accordingly, the two fungal isolates were selected for further identification and chromatographic characterization of their extracts.

### Morphological and molecular characterization

**Fig. 3:**
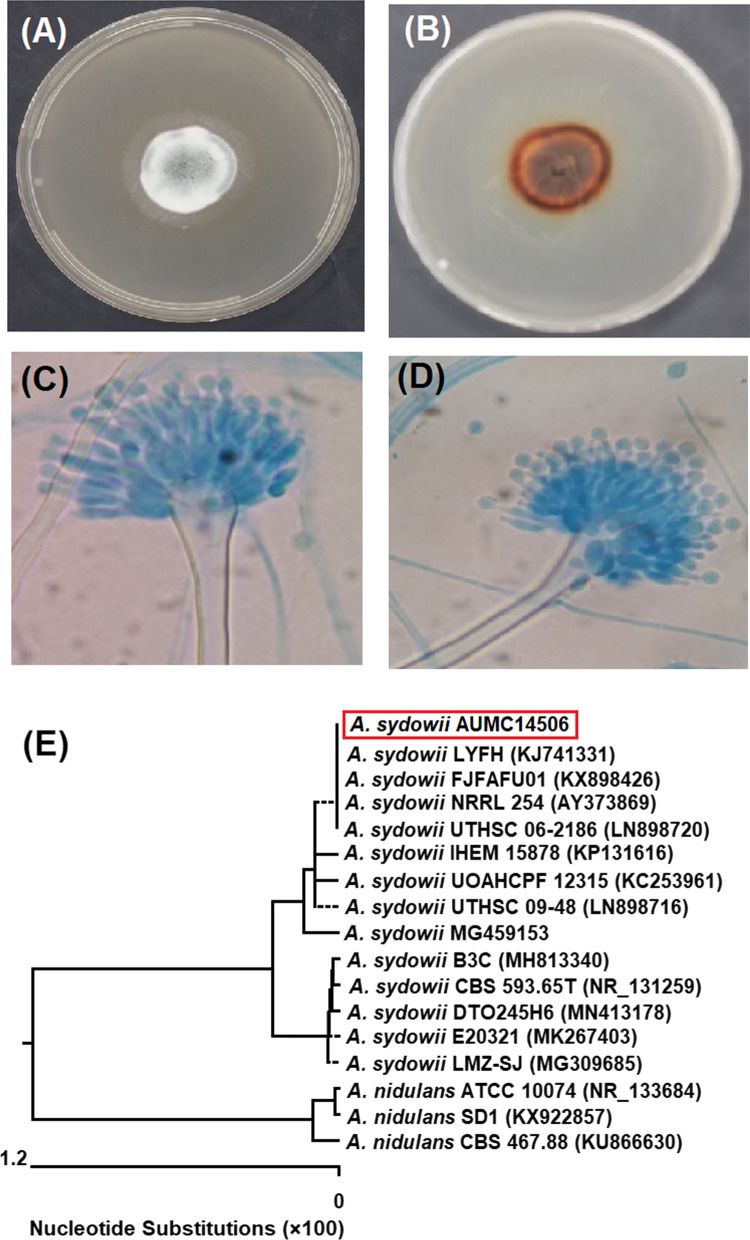
Morphological and molecular characteristics of *A. sydowii* AUMC14506. Colony growth was observed on Czapek Yeast autolystae agar after incubation for 10 days at 25 °C (**A**, **B**). Microscopic appearance of conidia and conidiophore (**C**, **D**). Phylogenetic tree of the fungal isolate (AUMC14506) and other closely related strains of *A. sydowii*, based on the ITS1-5.8S rRNA-ITS2 rDNA sequences (**E**)

Figure 3 showed the colony morphology of *Aspergillus sydowii* AUMC14506 grown on CYA-agar plates. The strain had a bluish green velutinous restricted colony in the right view at 25 °C after 10 days (Fig. 3A). Meanwhile, the reverse view showed a pale to orange brown color (Fig. 3B). Microscopic appearance showed vesicles, metulae, phialides and globose conidia (Fig. 3C, D). The sequence obtained from molecular characterization was deposited under the number MW092906 in the GenBank, then analyzed and a phylogenetic tree was developed. The obtained data (Fig. 3E) confirmed that *A. sydowii* AUMC14506 showed 99.47—100% identity with several strains of* A. sydowii* including the type strain CBS593.65 T. Strains of *A. nidulans* are included as outgroup species in the phylogenetic tree.

**Fig. 4:**
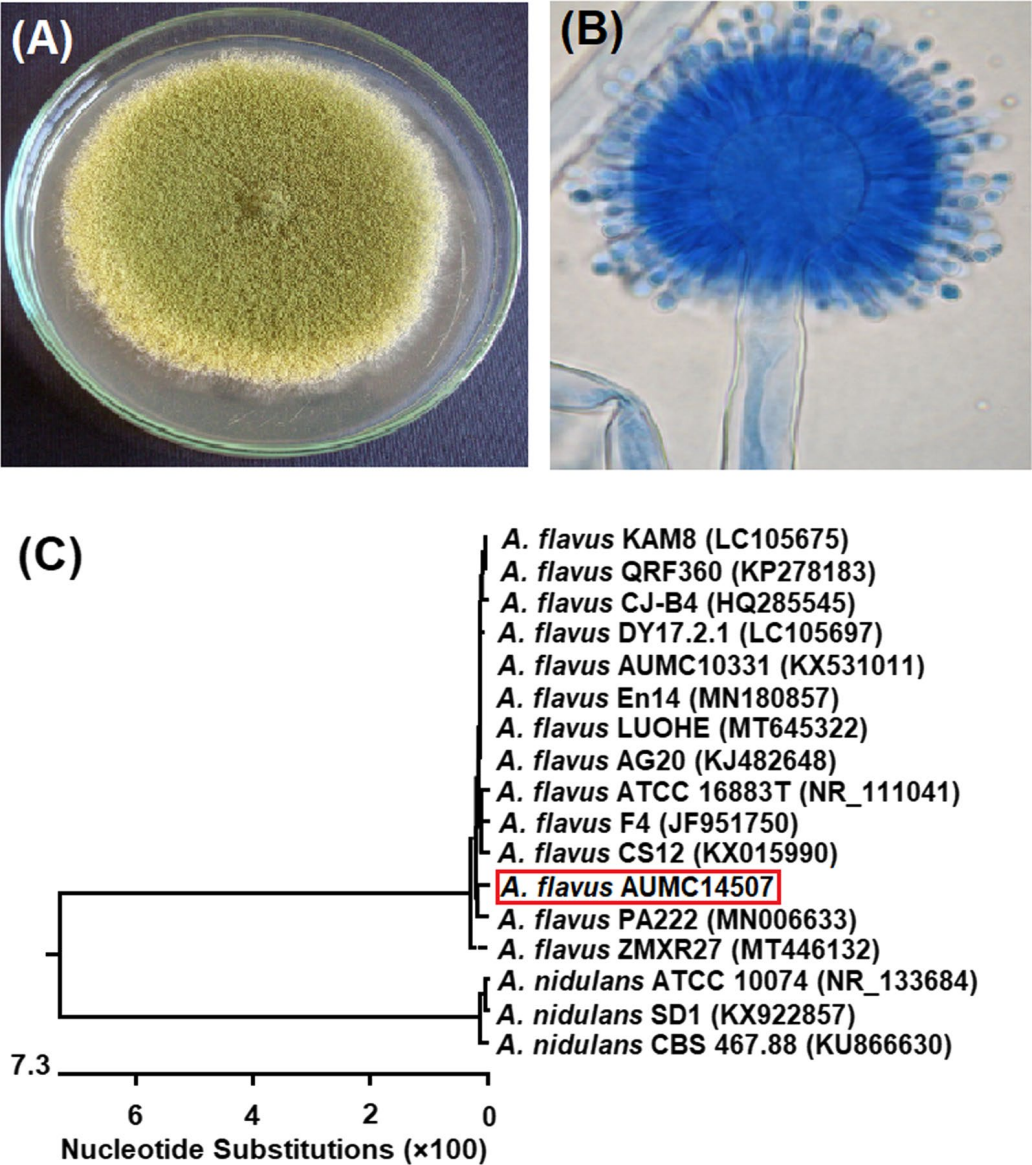
Morphological and molecular characteristics of *A. flavus* AUMC14507. Colony growth was observed on Czapek Yeast autolystae agar after incubation for 10 days at 25 °C (**A**). Microscopic appearance of conidia and conidiophore (**B**). Phylogenetic tree of the fungal isolate (AUMC14507) and other closely related strains of *A. sydowii*, based on the ITS1-5.8S rRNA-ITS2 rDNA sequences (**C**)

Figure 4 showed the colony morphology of *Aspergillus flavus* AUMC14507 grown on CYA-agar plates. The strain had a yellow green colony in the right view at 25 °C after 10 days (Fig. 4A). Meanwhile, microscopic appearance presented conidial head showing vesicle, metulae, phialides and rough-walled conidia (Fig. 4B). The sequence obtained from molecular characterization was deposited under the number MW092907 in the GenBank, then analyzed and a phylogenetic tree was developed. The obtained data (Fig. 4C) confirmed that *A. flavus* AUMC14507 showed 99.63%—100% identity with several strains of* A. flavus* including the Type strain ATCC16883T. Strains of *A. nidulans* are included as outgroup species in the phylogenetic tree.

### Identification of chemical constituents of the fungal extracts by GC–MS

**Fig. 5:**
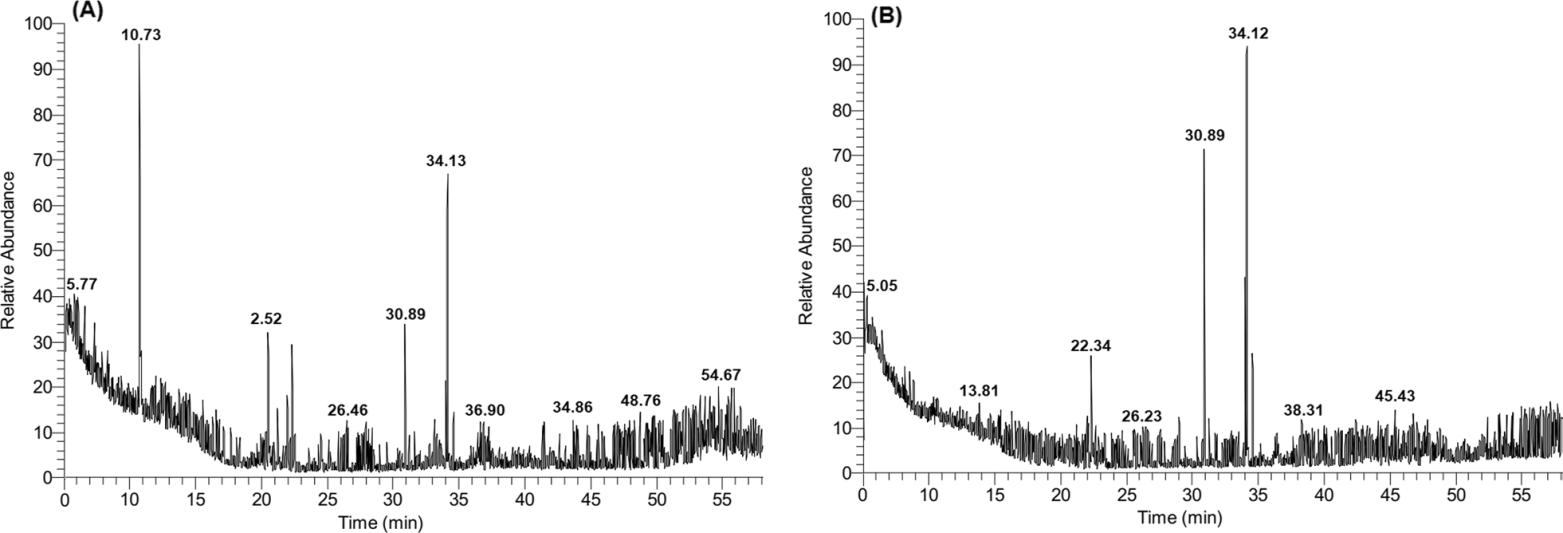
GC–MS chromatograms of crude extracts from fungal culture of *A. sydowii* **(A)** and *A. flavus* **(B)**. The methylene chloride extracts were evaporated and dissolved in 1 mL of HPLC-grade methanol and used under the conditions described in “Materials and methods” section

GC–MS analysis of the crude extracts of the *Aspergillus sydowii* AUMC14506 and *Aspergillus flavus* AUMC14507 revealed the presence of a wide array of compounds. The total peaks area of the detected compounds, the retention time, molecular weight, molecular formula, and structures of the detected compounds present in extracts are listed in Table 2 (for *A. sydowii*) and Table 3 (for *A. flavus*). The chromatograms are shown in Fig. 5 (5A, for *A. sydowii*) and (5B, for *A. flavus*). Forty compounds (1.00 to 9.00% percent peak area) were identified in fungal extract of *A. sydowii* (Table 2) where the major compounds were 9-Octadecenoicacid methyl ester (19.00%), ( +)-(1 s,2 s)-(2-phenylcyclopropyl) methanol (7.07%), Hexadecanoic acid,methyl ester (4.05%), 1-(3-Isobutyryl -bicyclo[1.1.1]pent-1-yl-)-2-methylpropan-1-one (3.62%), and 4,5,6,6a,10′,11-′hexahydrospiro{5′H-dibenzo[a,d]cycloheptene-5′,3(3aH)-[4,5,6]methenocyclopentapyrazole}(3.56%). Meanwhile, a total of forty compounds (1.05 to 18.08% percent peak area) were identified in *A. flavus* extract (Table 3) where the major compounds were 29-Octadecenoicacid,methyl ester, (E)-(CAS) (18.08%), Hexadecanoic acid, methyl ester (10.18%), 9,12-Octadecadienoic acid, methyl ester, (E,E)-(CAS) (4.90%) and 3,4-Dihydro-2H-1,5(3″-t-butyl) Benzodioxepine (4.12%).

**Table 2:** GC–MS analysis of the methylene chloride extract of *A. sydowii* AUMC14506

No	RT (min(	MW	MF	Area (%)	Detected compounds	Bioactivity	Reference
1	5.08	148	C_10_H_12_O	7.07	( +)-(1 s,2 s)-(2-phenylcyclopropyl) methanol	Antimicrobial	Bisht et al. (2010)
2	6.16	649	C_32_H_19_Cl_4_N_3_O_4_	1.20	4,5,6,7-Tetrakis(p-chlorophenoxy) 1,2-diiminoisoin Doline	Antitumor	Ol´shevskaya et al. (2014)
3	7.31	129	C_8_H_19_N	1.41	1-Butanamine,N-butyl-(CAS)		
4	9.93	44	C_2_H_4_O	1.06	Oxirane (CAS)	Antimicrobial, Anticancer	Thirunarayanan and Vanangamudi (2011)
5	12.68	648	C_44_H_56_O_4_	1.43	5,11,17,23-Tetra-t-butyl-25,26,27,28-tetrahydroxycalix-4-arene	Antimicrobial	Muneer et al. (2016)
6	12.94	692	C_44_H_44_N_4_O_4_	1.09	N,N′-Dicyclohexyl-1,7-dipyrrolidinylperylene-3,4:9,10-tetracarboxylic acid bisimide		
7	13.09	692	C_45_H_32_N_4_O_4_	1.06	8-Oxo-5,6-dihydro-6-(methoxycarbonyl)-5,10,15,20-tetraphenyl-8H-7- oxaprphyrin		
8	16.26	678	C_45_H_58_O_5_	1.17	7,13,19,25-Tetra-tert-butyl-27,28,29,30-tetrahydroxy-2,3-bishomo-3-oxacalix[4]arene	Antimicrobial	Muneer et al. (2016)
9	16.57	640	C_45_H_36_O_4_	1.10	5,10,10,11Tetra(4-methoxypheny)-10H-benzo[b]fluorene		
10	17.14	649	C_32_H_19_C_l4_N_3_O_4_	1.35	4,5,6,7-Tetrakis(p-chlorophenoxy)-1,2-diiminoisoindoline	Antitumor	Ol´shevskaya et al. (2014)
11	20.35	626	C_40_H_34_N_8_	1.76	Tetraethyl-phthalocyanine	Antitumor	Boyle et al. (1993)
12	20.53	208	C_13_H_20_O_2_	3.62	1-(3-Isobutyryl -bicyclo[1.1.1]pent-1-yl-)-2-methylpropan-1-one		
13	21.19	152	C_10_H_16_O	2.17	à-methyl-à-(2-propenyl)-1-cyclopentene-1-methanol	Antimicrobial	Bisht et al (2010)
14	21.98	102	C_5_H_10_O_2_	2.51	2- Furanmethanol,tetrahydro-(CAS)		
15	22.34	298	C_21_H_18_N_2_	3.56	4,5,6,6a,10′,11-'hexahydrospiro{5′H-dibenzo[a,d]cycloheptene- 5′,3(3aH)-[4,5,6]methenocyclopentapyrazole}		
16	27.43	539	C_35_H_57_NO_3_	1.12	N-Butyl,N-methyl-11-(3′-methoxy-17′á-hydroxy-1′,3′,5′(10′)-estratrien-15′(à,beta.)-yl)undecanamide		
17	27.63	661	C_45_H_63_N_3_O	1.32	Myrmicarin 661	Pharmacological	Snyder et al. (2010)
18	30.90	270	C_17_H_34_O_2_	4.05	Hexadecanoic acid,methyl ester (CAS)	Antibacterial	Astiti and Ramona (2021)
19	34.03	678	C_40_H_46_N_4_O_6_	2.20	2,3-diethyl-6,7-bis(2-methoxycarbonylethyl)-ç-(2-methoxycarbonylvinyl)-1,4,5,8-tetramethylporphyrin	Antibacterial	Sułek et al. (2020)
20	34.13	296	C_19_H_36_O_2_	19.0	9-Octadecenoicacid,methyl ester, (E)-(CAS)	Anticancer	Ahamed et al. (2020)
21	34.58	298	C_19_H_38_O_2_	1.30	Octadecanoic acid,methyl ester (CAS)	Cytotoxic	Atasever-Arslan et al. (2015)
22	37.14	694	C_48_H_62_N_4_	1.74	2,7,12,17-Tetraethyl-3,5:8,10:13,15:18,20-tetrakis (2,2-dimethylpropano) porphyrin	Antibacterial	Amos-Tautua et al. (2020)
23	46.74	606	C_36_H_38_O_5_	1.06	Methyl phaeophorbide-a	Antioxidant	Yoon et al. (2011)
24	47.15	692	C_44_H_44_N_4_O_4_	1.05	N,N′-Dicyclohexyl-1,7-dipyrrolidinylperylene-3,4:9,10- tetracarboxylic acid bisimide		
25	47.88	661	C_45_H_63_N_3_O	1.09	Myrmicarin 661	Pharmacological	Snyder et al (2010)
26	48.18	669	C_41_H_29_Cl_2_NO_4_	1.00	2,4-Di(4-chlorobenzoyl)3,5-diphenyl-6-(4- methoxyphenyl) aniline acetanilide		
27	48.28	633	C_37_H_44_ClNO_6_	1. 20	PENITREM A	Anticancer Antimicrobial	Sallam et al. (2013) Amos-Tautua et al. (2020)
28	48.76	648	C_35_H_38_Cl_2_N_4_O_4_	1.29	2,4bis-(á-chloroethyl)-6,7-bis[ámethoxycarbonylethyl]-1,3,5- trimethylporphyrin		
29	49.08	611	C_38_H_33_N_3_O_5_	1.01	N-Cyclohexyl-1,7-dipyrrolidinylperylene-3,4:9,10-tetracarboxylicacid3,4-anhydride-9,10-imide		
30	49.47	277	C_19_H_35_N	1.04	3,5Bis(cyclohexylmethyl)piperidine	Antioxidant	Manjusha et al. (2018)
31	49.65	603	C_33_H_46_ClNO_7_	1.32	Milbemycin b,13-chloro-5-demethoxy-28-deoxy-6,28-epoxy -5-(hydroxyimino)-25-(1-methylethyl)-,(6R,13R,25R)-		
32	51.41	711	C_43_H_37_NO_9_	1.85	3,6-Diphenyl-3,6-(hydroxyimino)-4,5-(2,2'-diphenylene) tricyclo [6.2.1.0(2,7)]undeca-3,5-diene-9,10-(E)-dicarboxylic Acid diacetoxy methyl ester		
33	51.93	678	C_45_H_58_O_5_	1.37	7,13,19,25-Tetra-tert-butyl-27,28,29,30-tetrahydroxy-2,3-bishomo-3-oxacalix[4]arene		
34	52.22	660	C_20_Cl_12_	1.16	Dodecachloroperylene		
35	52.73	616	C_32_H_40_O_12_	1.21	Anodendroside E 2,monoacetate	Antimicrobial	Abdel-Rahman et al. (2019)
36	52.80	616	C_32_H_40_O12	1.73	Anodendroside E 2,Monoacetate	Antimicrobial	Abdel-Rahman et al. (2019)
37	53.28	536	C_28_H_40_O_10_	1.29	9-Desoxo-9-xacetoxy-3,8,12-tri-O-acetylingol	Antibacterial	Shareef et al. (2016)
38	53.39	694	C_48_H_62_N_4_	1.59	2,7,12,17-Tetraethyl-3,5:8,10:13,15:18,20-tetrakis(2,2-dimethylpropano) Porphyrin	Antibacterial	Sułek et al. (2020
39	54.67	542	C_40_H_62_	1.37	Phytofluene	Antimicrobial	Engelmann et al. (2011)
40	54.95	582	C_30_H_42_N_6_O_6_	1.28	2,4,6-Tris(1-(2-methoxyc arbonylpyrrolidin-1-yl)propen-2-yl)-1,3,5-triazine	Antiinfammatory	El-Reedy and soliman (2020)

**Table 3:** GC–MS analysis of the methylene chloride extract of *A. flavus* AUMC14507

No	RT (min(	MW	MF	Area (%)	Detected compounds	Bioactivity	Reference
1	5.05	324	C_19_H_20_N_2_O_3_	4.63	20-Deethyl-17-oxovincadifformine	Cytotoxic, Antimicrobial	Zhang et al. (2014)
2	5.23	129	C_8_H_19_N	1.22	N,N-Bis (isobutyl)amine	Antitumor, Antimicrobial,	Kumar et al. (2018)
3	5.30	116	C_5_H_8_O_3_	1.44	( +)-(S)-5-Hydroxymethyloxolan-2-one		
4	6.43	84	C_4_H_8_N_2_	1.10	Acetonitrile, (dimethylamino)- (CAS)		
5	10.37	386	C_18_H_15_ClN_4_O_4_	1.13	3-(p-Ethoxyphenyl)-4-[5-(2-chlorophenyl)-dealta.(2)-1,2,4-oxadiazolin-3-yl]sydnone	Antimicrobial	Mallur and Badami (2000)
6	10.65	572	C_37_H_32_O_6_	1.05	(-)-2,2′,3,3′-Tetraethyl-(8-hydroxy-8-methoxy)-7,7′-bi(phenanthrene-1,4-dione)	Cytotoxicity, Antimicrobial	Kovács's et al. (2008)
7	15.94	696	C_51_H_68_O	1.11	Bis(3,6-di-tert-butyl-1-azulenyl)(3,5-ditert-butyl-4-hydroxyphenyl)methane	Antibacterial, Antioxidant	Praveen et al. (2015)
8	16.86	692	C_44_H_44_N_4_O_4_	1.19	N,N-'Dicyclohexyl-1,7-dipyrrolidinylperylene-3,4:9,10-tetracarboxylicacidbisimide		
9	17.87	658	C_44_H_42_N_4_O_2_	1.10	2,9-Bis(5-tert-butyl-2-methoxy-3-pyridylphenyl)-1,10-phenanthroline		
10	17.94	696	C_40_H_56_O_10_	1.24	Nephthoside -1,2′,3′,4′-Tetraacetate		
11	18.52	656	C_36_H_52_N_2_O_9_	1.57	Jiufengdine	Drug lead	Zhou et al. (2004)
12	19.40	698	C_48_H_34_N_4_O_2_	1.65	Cis-5,10-bis(4-formylphenyl)-15,20-di(4′′-tolyl) porphyrine	Photodynamic therapy of tumor	Lamarche and Francois (2002)
13	21.17	659	C_34_H_45_NO_12_	1.10	3-Acetoy-8-deacetoxy-N,19-seco-22-nor-17-(1,2-oxazocyclopropan-1-yl) yunaconitine	Antiinfammatory, Antineoplastic	Zhang et al. (2020)
14	22.00	760	C_48_H_56_O_8_	1.37	Methoxychromene precocene tetramer		
15	22.05	58	C_4_H_10_	1.81	Butane (CAS)		
16	22.34	206	C_13_H_18_O_2_	4.12	3,4-Dihydro-2H-1,5(3′′-t-butyl) Benzodioxepine	Antioxidant	Zhang et al. (2014)
17	22.86	666	C_26_H_16_Cl_6_O_8_	1.11	Russuphelol	Cytotoixic	Laus (2001)
18	24.48	636	C_18_Cl_12_	1.06	Dodecachloro-3,4-benzo Phenanthrene	Anticancer	Li et al. (2006)
19	26.43	60	C_8_H_17_NO	1.40	2-[N-(tButylamino)]-2-methylpropanal		
20	26.68	618	C_39_H_54_O_6_	1.24	2-Hydroxy-3-methoxy-6,7,10,11-tetrapentyloxytriphenylene		
21	30.89	270	C_17_H_34_O_2_	10.18	Hexadecanoic acid, methyl ester (CAS)	Antibacterial	Astiti and Ramona (2021)
22	31.62	678	C_36_H_40_Cl_2_N_4_O_5_	1.08	2,4-bis(2-chloroethyl)-8-h ydroxymethyl)-6,7-bis[2-(methoxycarbonyl) ethyl]-1,3,5-trimethylporphyrin	Photodynamic therapy of tumor	Lamarche et al. (2002)
23	32.77	111	C_5_H_5_NO_2_	1.06	3-Deazauracil	Antiviral,Anticancer	Ogilvie et al. (1984)
24	34.03	294	C_19_H_34_O_2_	4.90	9,12-Octadecadienoic acid, methyl ester, (E,E)-(CAS)		
25	34.12	296	C_19_H_36_O_2_	18.08	9-Octadecenoicacid,methyl ester, (E)-(CAS)	Anticancer	Ahamed et al. (2020)
26	34.58	340	C_22_H_44_O_2_	3.04	Heneicosanoic acid, methyl ester (CAS)	Antimicrobial Antioxidant	Salem et al. (2014)
27	38.46	729	C_37_H_47_NO_14_	1.13	N-De-ethylyunaconitineimine N-oxide		
28	39.12	670	C_48_H_38_N_4_	1.05	2-Butyl-5,10,15,20- tetraphenylporphyrin		
29	41.07	486	C_27_H_38_N_2_O_6_	1.37	4,25-Secoobscurinervan4-ol,25-ethyl-15,16-dimethoxy-,25 acetate,(4á,22à)-		
30	41.65	144	C_9_H_20_O	1.34	3-Ethyl-2-heptanol		
31	42.06	126	C_7_H_14_N_2_	1.07	6-Cyanohexanamine		
32	42.14	378	C_22_H_22_N_2_O_4_	1.12	Isopropyl4,6-dioxo-3,5-diphenyl-perhydropyrrolo[3,4-c] Pyrrole-1-carboxylate		
33	42.40	696	C_40_H_56_O_10_	1.18	Nephthoside -1,2′,3′,4′-Tetraacetate	Cytotoxic Antioxidant	Sunassee and Davies-Coleman (2012)
34	42.59	692	C_44_H_44_N_4_O_4_	1.12	N,N′-Dicyclohexyl-1,7-dipyrrolidinylperylene-3,4:9,10-tetracarboxylic acid bisimide		
35	43.18	616	C_32_H_40_O_12_	1.06	Anodendroside E 2,monoacetate	Antimicrobial	Abdel-Rahman et al. (2019)
36	44.41	633	C_37_H_44_ClNO_6_	1.21	PENITREM A	Antimicrobial Anticancer	Sallam et al. (2013) Amos-Tautua et al. (2020)
37	44.75	692	C_44_H_44_N_4_O_4_	1.15	N,N′ –Dicyclohexyl-1,7-dipYrrolidinylperylene-3,4:9,10-tetracarboxylic acid bisimide		
38	47.81	676	C_36_H_60_N_4_O_8_	1.04	Bis(3,6,9,12-tetraoxapentaethylene)crowno-N,N,N′,N′-tetramethyl-p- phanediamine		
39	53.65	489	C_27_H_39_NO_7_	1.69	3-Acetoxy-8,9-dehydro-12,13-secopseudaconin-14-one		
40	54.32	660	C_32_H_40_O_12_	1.61	Anodendroside E 2,monoacetate (CAS)	Antimicrobial	Abdel-Rahman et al. (2019)

### Improving the antifungal, antibacterial, cytotoxic, and antioxidant potentials *A. sydowii* and *A. flavus* by gamma irradiation

Several gamma irradiation doses ranged from 250 to 4000 Gy used to study their effects on enhancing bioactivities of *A. sydowii* and *A. flavus*. Generally, remarkable feature of the obtained results is the dose-related behavior of the influence of gamma irradiation on the recorded activities either antimicrobial (Tables 4 and 5), antioxidant, or anticancer (Table 6) for both strains. The recorded data on the estimated bioactivities further indicated that 1000 Gy was the most proper dose for attaining the highest rates of the antifungal, antibacterial, anticancer and antioxidant activities for both strains. When compared to the control (non-irradiated), significant differences (*P* ≤ *0.05*) in the recorded values were observed for both strains in all the measured activities (Tables 4, 5, and 6).

**Table 4:** Effect of different doses of gamma irradiation on the antifungal activity (expressed as diameter of inhibition zone in mm) of *A. sydowii* AUMC14506 and *A. flavus* AUMC14507

Gamma irradiation dose	*A. sydowii *AUMC14506		*A. flavus* AUMC14507	
(Gy)	*A. brasiliensis*	*C. albicans*	*A. brasiliensis*	*C. albicans*
0.00 (C)	14.33 ± 0.00^ cd^	18.00 ± 1.00^c^	12.67 ± 2.08^c^	16.33 ± 0.58^ c^
250	16.67 ± 1.06^ cd^	20.00 ± 1.66^c^	14.33 ± 1.66^c^	18.33 ± 2.05^c^
500	22.33 ± 1.52^b^	25.67 ± 1.66^b^	20.00 ± 0.58^b^	24.88 ± 1.15^b^
1000	28.33 ± 1.77^a^	31.33 ± 2.56^a^	26.33 ± 1.54^a^	29.00 ± 2.57^a^
2000	11.67 ± 1.66^d^	12.33 ± 1.78^d^	10.00 ± 1.00^c^	10.57 ± 1.15^d^
4000	0.00^e^	0.00^e^	0.00^d^	0.00^e^

Calculated mean is for triplicate measurements from two independent experiments ± SD

^a−e^ means with different superscripts in the same column for each nanoparticle are considered statistically different (LSD test, *P* ≤ 0.05)

**Table 5:** Effect of different doses of gamma irradiation on the antibacterial activity (expressed as diameter of inhibition zone in mm) of *A. sydowii* AUMC14506 and *A. flavus* AUMC14507

Gamma irradiation dose	*A. sydowii* AUMC14506		*A. flavus* AUMC14507	
(Gy)	*E. coli*	*S. aureus*	*E. coli*	*S. aureus*
0.00 (C)	18.33 ± 1.53^c^	16.33 ± 1.00^c^	17.33 ± 0.58^c^	15.67 ± 1.67^c^
250	20.67 ± 0.58^c^	18.67 ± 1.67^c^	19.33 ± 1.58^c^	19.67 ± 2.05^c^
500	25.33 ± 1.53^b^	25.67 ± 1.05^b^	25.00 ± 1.00^b^	26.33 ± 0.58^b^
1000	34.00 ± 1.00^a^	33.67 ± 2.56^a^	31.67 ± 2.05^a^	32.67 ± 2.57^a^
2000	12.33 ± 0.58^d^	11.00 ± 1.78^d^	12.00 ± 1.00^d^	11.33 ± 1.06^d^
4000	0.00^e^	0.00^e^	0.00^e^	0.00^e^

Calculated mean is for triplicate measurements from two independent experiments ± SD, ^a−e^ means with different superscripts in the same column for each nanoparticle are considered statistically different (LSD test, *P* ≤ 0.05)

**Table 6:** Effect of different doses of gamma irradiation on the antioxidant (% of DPPH scavenging) and cytotoxic (% of viability) activities of *A. sydowii* AUMC14506* and A. flavus* AUMC14507

Gamma irradiation dose	DPPH scavenging (%)		Viability (%)	
(Gy)	AUMC14506	AUMC14507	AUMC14506	AUMC14507
0.00 (C)	14.65 ± 1.32^d^	13.87 ± 1.78^d^	31.55 ± 1.32^b^	40.87 ± 1.11^b^
250	22.55 ± 1.87^c^	22.52 ± 1.03^c^	28.01 ± 1.74^c^	36.41 ± 1.86^c^
500	28.09 ± 2.11^b^	27.89 ± 2.43^b^	14.99 ± 2.06^d^	22.87 ± 4.11^d^
1000	53.67 ± 3.65^a^	48.87 ± 2.11^a^	09.96 ± 0.41^e^	09.41 ± 5.92^e^
2000	10.55 ± 1.83^d^	11.06 ± 1.01^d^	45.57 ± 2.33^a^	43.05 ± 1.23^a^
4000	0.00^e^	0.00^e^	0.00^f^	0.00^f^

Calculated mean is for triplicate measurements from two independent experiments ± SD, ^a−f^ means with different superscripts in the same column for each nanoparticle are considered statistically different (LSD test, *P* ≤ 0.05)

## Discussion

Endophytic fungi are currently suggested as the most promising sources for new metabolites (El-Sayed 2021) with diverse range of bioactivities that will surely open the way for several medical, agricultural, and industrial applications. In this context, metabolites derived from fungal endophytes are gaining considerable attention to be used as novel antimicrobial agents. Thus, the aim of this study lies in an even less understood research area with significant unexploited potential: bioactive metabolites from fungal endophytes of some certain plants in Egypt. As a first step, 69 endophytic strains were isolated from eleven different plant species. The isolated endophytes were found to belong to eight genera as follows: *Aspergillus*, *Acremonium*, *Cladosporium*, *Trichoderma, Lasiodiplodia, Fusarium*, *and Alteranria*. The isolated strains were then cultivated in PDA plates and PD broth (pH 6.0), then extracted and tested for their antifungal (against *C. albicans* and *A. brasiliensis*), antibacterial (*S. aureus* and *E. coli*), cytotoxic (against MCF-7 breast cancer cell lines), and free DPPH-scavenging potentials. The screening profile of the fungal extracts revealed that 12 fungal strains shown bioactive potential with vary degrees. Surprisingly, two fungal strains, amongst the 69 strains, displayed all the tested activities of antifungal, antibacterial, cytotoxic, and antioxidant potentials, thus they were selected. Morphological examination of the two fungal strains maintained on the CYA agar (grown at 25 °C for 10 days) were identical with the characteristics concerning the identification of *A. sydowii* and *A. flavus* (Moubasher 1993). As such, data obtained from the molecular studies of these strains confirmed the high conformity with their closely related fungi.

In this study, identification of chemical constituents of the methanolic extracts of the two fungal strains was accomplished by GC–MS. The obtained results confirmed presence of several well-known bioactive compounds produced by the two fungal strains. In fungal extract of *A. sydowii*, the major compounds were 9-Octadecenoicacid methyl ester (Anticancer, Ahamed et al. 2020), ( +)-(1 s,2 s)-(2-phenylcyclopropyl) methanol (Antimicrobial, Bisht et al. 2010), Hexadecanoic acid,methyl ester (Antimicrobial, Astiti and Ramona 2021), 1-(3-Isobutyryl -bicyclo[1.1.1]pent-1-yl-)-2-methylpropan-1-one (unreported), and 4,5,6,6a,10′,11-′hexahydrospiro{5′H-dibenzo[a,d]cycloheptene-5′,3(3aH)-[4,5,6]methenocyclopentapyrazole}. Moreover, several bioactive compounds were detected in smaller amounts. Table 2 presented all the reported compounds and their bioactivity. Meanwhile, *A. flavus* extract showed major compounds of 9-Octadecenoicacid,methyl ester, (E)-(CAS) (Anticancer, Ahamed et al. 2020), Hexadecanoic acid, methyl ester (Antimicrobial, Astiti and Ramona 2021), 9,12-Octadecadienoic acid, methyl ester, (E,E)-(CAS) (Anticancer, Ahamed et al. 2020), and 3,4-Dihydro-2H-1,5(3″-t-butyl) Benzodioxepine (Antioxidant, Zhang et al. 2014). Moreover, several bioactive compounds were detected in smaller amounts. Table 3 presented all the reported compounds and their bioactivity.

Here, the presented study was extended to evaluate the use of gamma irradiation for improving the achieved bioactivities of *A. sydowii* and *A. flavus.* Our results confirmed the positive role of gamma rays at 1000 Gy for achieving the highest values of the antifungal, antibacterial, cytotoxic, and antioxidant activities for both strains. In agreement with these results, the same dose of gamma rays (1000 Gy) was used for improving titers of the cardiac glycoside digoxin produced by *Epicoccum nigrum* (El-Sayed et al. 2020c) with a recorded five-fold increase higher than the control cultures. Moreover, the most 500 Gy of gamma rays was used for maximum production of the acetylcholinesterase inhibitor HupA from *A. brassicae* cultures (Zaki and El-Sayed 2021). Also, titers of the anticancer drug taxol was successfully intensified following gamma radiation of *Alternaria tenuissima* and *Aspergillus fumigatus* at 750 Gy (El-Sayed et al. 2019b, 2019c). In another connection, lower doses of gamma rays (5–250 Gy) *Alternaria tenuissima* cultures to improve the yield of sugars and proteins from (Geweely and Nawar 2006). Generally, gamma radiation is a powerful energetic ionizing radiation (El-Sayed et al. 2019a; 2020a). Exposure of living cell to this type of radiation at certain doses can initiate mutations through several mechanisms such as the DNA repair of genes (El-Sayed et al. 2020b; d). Most recently, mutagenesis by gamma rays was highly recommended in this regard to improve the target microbial strains thereby intensifying their producing capabilities (El-Sayed et al. 2020e, f; Zaki et al. 2020; and references therein).

In summary, two promising endophytic fungal strains showed antifungal, antibacterial, cytotoxic, and antioxidant potentials. The strains were identified as *Aspergillus sydowii* AUMC14506 and *A. flavus* AUMC14507, based on morphological and molecular studies. Chemical constituents present in extracts of the two strains were identified by GC-Mass analyses. Morevover, all the achieved bioactivities of the two strains were significantly intensified using 1000 Gy gamma rays. These findings strongly recommend the bioprospection of endophytic fungi as promising sources of bioactive metabolites with antimicrobial, anticancer, and antioxidant potentials.

## Data Availability

All data generated or analyzed during this study are included in this published article.
